# Supplementary material for: Environmentally Safe Biosynthesis of Gold Nanoparticles Using Plant Water Extracts
Source: Nanomaterials (Basel). 2021 Aug 10;11(8):2033. doi: 10.3390/nano11082033 (PMC8400837; doi:10.3390/nano11082033)
Supplement: Supplementary file 1 [file nanomaterials-11-02033-s001.zip › nanomaterials-1313929-supplementary.pdf]

Online Supporting Materials

Environmentally safe biosynthesis of gold nanoparticles using plant water extracts

Mohadeseh Hassanisaadi[1], Gholam Hosein Shahidi Bonjar[1, 2, *], Abbas Rahdar[3], Sadanand Pandey[4, *], Akbar Hosseinipour[1], Roohollah Abdolshahi[5]

[1] Department of Plant Protection, Shahid Bahonar University of Kerman, Postal Code: 7618411764, Kerman, Iran; mhassani@agr.uk.ac.ir (M.H.); Hosseini@uk.ac.ir (A.H.)

[2] Medical Mycology and Bacteriology Research Center, Kerman University of Medical Sciences, Kerman 7616913555, Iran

[3] Department of Physics, Faculty of Science, University of Zabol, Postal Code: 538-98615, Zabol, Iran; a.rahdar@uoz.ac.ir (A.R.)

[4] Department of Chemistry, College of Natural Science, Yeungnam University, 280 Daehak-Ro, Gyeongsan, Gyeongbuk, 38541, Republic of Korea

[5]Department of Agronomy and Plant Breeding, Shahid Bahonar University of Kerman, Postal Code: 7618411764, Kerman, Iran; abdoshahi@uk.ac.ir (R.A.)

* Correspondence: shahidi@uk.ac.ir (G.H.S.B); sadanand.au@gmail.com ; spandey@ynu.ac.kr (S.P.)

Table S1. Screening of medicinal plants used in folkloric medicine of Middle East countries for bio-reduction Au^{3+} to Au^{0}. Water extracts of plant organs of 109 plant species belonging to 54 plant families evaluated in this survey.

No.	Scientific name	Family	VN[1]	PP[2]	CI[3]	CO[4]
1	*Amaranthus* sp.	Amaranthaceae	AMAR55	Se	3	V
2	*Rhus* sp.	Anacardiaceae	ANAC80	Fr	2	G
3	*Pistacia lentiscus*	Anacardiaceae	ANAC120	Gu	1	V
4	*Pistacia atlantica*	Anacardiaceae	ANAC106	Fr	2	G
5	*Carum carvi*	Apiaceae	APIA6	Fr	0	-
6	*Anethum graveolens*	Apiaceae	APIA10	Le	1	G
7	*Foeniculum vulgare*	Apiaceae	APIA31	Fr	2	G
8	*Heracleum persicum*	Apiaceae	APIA43	Fr	2	G
9	*Trachyspermum ammi*	Apiaceae	APIA62	Fr	3	G
10	*Anethum graveolens*	Apiaceae	APIA98	Fr	3	RP
11	*Pastinaca sativa*	Apiaceae	APIA105	Ro	3	RP
12	*Cuminum cyminum*	Apiaceae	APIA117	Fr	3	RP
13	*Areca catechu*	Arecaceae	AREC34	Fr	4	RP
14	*Cocos nucifera*	Arecaceae	AREC75	Fr	1	V
15	*Cichorium intybus*	Asteraceae	ASTE2	Se	1	G
16	*Achillea millefolium*	Asteraceae	ASTE7	Ab	1	V
17	*Artemisia cina*	Asteraceae	ASTE18	Se	1	PG
18	*Matricaria chamomilla*	Asteraceae	ASTE22	Fl	3	RP
19	*Lactuca sativa*	Asteraceae	ASTE41	Le	3	V
20	*Artemisia dracunculus*	Asteraceae	ASTE58	Le	3	G
21	*Pyrethrum roseum*	Asteraceae	ASTE60	Fr	3	V
22	*Carthamus tinctorius*	Asteraceae	ASTE70	Fl	1	V
23	*Berberis vulgaris*	Berberidaceae	BERB73	Fr	0	G
24	*Echium amoenum*	Boraginaceae	BORA23	Fl	2	V
25	*Cordia myxa*	Boraginaceae	BORA 46	Fr	0	-

26	*Caccinia macranthera*	Boraginaceae	CACC64	Le	4	V
27	*Nasturtium officinalis*	Brassicaceae	BRAS49	Ab	1	G
28	*Lepidium sativum*	Brassicaceae	BRAS66	Se	1	G
29	*Descurainia sophia*	Brassicaceae	BRAS74	Se	0	G
30	*Alyssum* sp.	Brassicaceae	BRAS107	Se	0	G
31	*Sinapis alba*	Brassicaceae	BRAS119	Se	0	G
32	*Cannabis sativa*	Canabinaceae	CANA113	Se	1	V
33	*Eugenia caryophyllata*	Caryophyllaceae	CARY47	Fl	2	G
34	*Terminalia chebula*	Combretaceae	COMB42	Fr	2	V
35	*Equisetum Arvense*	Equiseraceae	EQUI77	Ab	3	RP
36	*Senna* sp.	Fabaceae	FABA88	Le	2	RP
37	*Quercus* sp.	Fagaceae	FAGA14	Fr	0	G
38	*Fraxinus excelsior*	Fraxinaceae	FRAX111	Fr	2	G
39	*Fumaria officinalis*	Fumariaceae	FUMA9	Ab	0	-
40	*Erodium* sp.	Geraniaceae	GERA3	Ab	2	G
41	*Juglans regia*	Juglandaceae	JUGL15	Ba	4	RP
42	*Mentha* sp.	Lamiaceae	LAMI1	Le	1	G
43	*Mentha pulegium*	Lamiaceae	LAMI13	Le	1	G
44	*Melissa officinalis*	Lamiaceae	LAMI16	Ab	1	PG
45	*Lavandula* sp.	Lamiaceae	LAMI21	Ab	2	PG
46	*Teucrium polium*	Lamiaceae	LAMI24	Ab	1	G
47	*Zataria multiflora*	Lamiaceae	LAMI26	Le	1	G
48	*Artemisia absinthium*	Lamiaceae	LAMI30	Fl	3	RP
49	*Ocimum basilicum*	Lamiaceae	LAMI32	Fl	3	PG
50	*Stachys lavandulifolia*	Lamiaceae	LAMI33	Ab	1	V
51	*Hyssopus Officinalis*	Lamiaceae	LAMI48	Fl	3	RP
52	*Ocimum basilicum*	Lamiaceae	LAM-79	Se	0	-

53	*Origanum majorana*	Lamiaceae	LAMI90	Ab	1	G
54	*Cinnamomum zelanicum*	Lauraceae	LAUA59	Ba	3	RP
55	*Laurus nobilis*	Lauraceae	LAUA97	Le	3	RP
56	*Medicago sativa*	Leguminosae	LEGU36	Le	2	G
57	*Astragalus gossypinus*	Leguminosae	LEGU54	Gu	0	-
58	*Cassia Fistula*	Leguminosae	LEGU72	Fr	2	BG
59	*Trigonella foenum-graecum*	Leguminosae	LEGU84	Le	1	G
60	*Arachis hypogaea*	Leguminosae	LEGU93	Se	1	V
61	*Alhagi maurorum*	Leguminosae	LEGU99	Gu	1	V
62	*Astragalus adscendens*	Leguminosae	LEGU103	Gu	1	G
63	*Astragalus fasciculifolius*	Leguminosae	LEGU108	Gu	3	RP
64	*Medicago sativa*	Leguminosae	LEGU109	Se	2	V
65	*Allium stipitatum*	Liliaceae	LILI8	Bu	1	V
66	*Allium cepa*	Liliaceae	LILI27	Bu	1	G
67	*Allium sativum*	Liliaceae	LILI40	Bu	1	G
68	*Colchicum* sp.	Liliaceae	LILI56	Bu	2	RP
69	*Allium schoenoprasum*	Liliaceae	LILI85	Le	2	V
70	*Allium schoenoprasum*	Liliaceae	LILI92	Se	1	G
71	*Linum usitatissimum*	linaceae	LILI53	Se	1	G
72	*Crocus sativus*	lridaceae	IRID110	Sm	3	PG
73	*Malva sylvestris*	malvaceae	MALV57	Fl	0	-
74	*Myristica fragrans*	Myrsticaceae	MYRS116	Fr	2	RP
75	*Myrtus communis*	Myrtaceae	MYRT35	Le	2	G
76	*Myrtus communis*	Myrtaceae	MYRT68	Fr	4	PG
77	*Eucalyptus globulus*	Myrtaceae	MYRT86	Le	2	G
78	*Olea europaea*	Oleaceae	OLEA100	Le	1	G
79	*Orchis* sp.	Orchidaceae	ORCH115	Ro	0	-

80	*Papaver somniferum*	Papaveraceae	PAPA86	Se	2	V
81	*Sesamum indicum*	Pedaliaceae	PEDA37	Se	1	V
82	*Piper nigrum*	Piperaceae	PIPE39	Fr	3	RP
83	*Piper nigrum*	Piperaceae	PIPE81	Fr	2	RP
84	*Plantago ovata*	Plantaginaceae	PLAN38	Se	0	-
85	*Zea mays*	Poaceae	POAC25	Se	1	G
86	*Oryza sativa*	Poaceae	POAC45	Gl	0	-
87	*Oryza sativa*	Poaceae	POAC82	Se	1	G
88	*Rheum ribes*	Polygonaceae	POLY65	Le	1	G
89	*Rumex alpinus*	Polygonaceae	POLY76	Fr	3	RP
90	*Rheum palmatum*	Polygonaceae	POLY114	Rh	1	G
91	*Portulaca oleracea*	Portulacaceae	PORT12	Se	1	G
92	*Punica granatum*	Punicaceae	PUNI71	Fl	1	G
93	*Ranunculus* sp.	Ranunculaceae	RANU63	Ab	1	G
94	*Nigella sativa*	Ranunculaceae	RANU78	Se	3	V
95	*Ziziphus zizyphus*	Rhamnaceae	RHAM69	Fr	3	PG
96	*Ziziphus spina christi*	Rhamnaceae	RHAM87	Le	3	RP
97	*Rosa damascena*	Rosaceae	ROSA5	Fl	2	RP
98	*Cydonia oblonga*	Rosaceae	ROSA11	Fr	1	RP
99	*Amygdalus communis*	Rosaceae	ROSA94	Se	1	RP
100	*Prunus cerasus avium*	Rosaceae	ROSA104	Fs	1	V
101	*Rubia Tinctorum*	Rubiaceae	RUBI51	Fr	2	PG
102	*Coffee arabica*	Rubiaceae	RUBI112	Se	2	G
103	*Citrus aurantifulia*	Rutaceae	RUTA4	Fr	2	G
104	*Citrus aurantium*	Rutaceae	RUTA28	Fl	1	G
105	*Ruta graveolens*	Rutaceae	RUTA95	Fr	2	G
106	*Spinacia oleracea*	Salsolaceae	SALA61	Le	0	-

107	*Atropa belladonna*	Solanaceae	SOLA17	Fr	2	PG
108	*Capsicum annuum*	Solanaceae	SOLA50	Fr	2	RP
109	*Camellia sinensis*	Teaceae	TEAC83	Le	2	G
110	*Camellia sinensis*	Teaceae	TEAC118	Pe	1	PG
111	*Urtica dioica*	Urticaceae	URTI19	Le	3	V
112	*Valeriana officinalis*	Valerianaceae	VALE20	Ab	1	V
113	*Vitis vinifera*	Vitaceae	VITA91	Le	1	G
114	*Zingiber officinale*	Zingiberaceae	ZING44	Rh	2	G
115	*Elettaria cardamomum*	Zingiberaceae	ZING67	Fr	2	G
116	*Elettaria cardamomum*	Zingiberaceae	ZING96	Fr	2	RP
117	*Tribulus terrestris*	Zygophylaceae	ZYGO29	Fr	1	G

[1] VN: Voucher number of plants stored in Laboratory of Plant Systematic, College of Agriculture, Shahid Bahonar University of Kerman, Iran; [2] PP: Part plants used for biosynthesis of AuNPs (Ab: above-ground parts, Ba: bark, Bu: bud, Fl: flower, Fr: fruit, Fs: fruit stalk, Gl: glumes, Le: leaf, Pe: petioles, Rh: rhizome, Ro: root, Se: seeds, Sm: flower stamen and Wh: whole plant); [3] CI: Color intensity (0: no reaction, 1: slight, 2: moderate, 3: intense, and 4:very intense); [4] CO: Changed color by reduction of Au^{3+} to Au^{0} (R: red, G: gray, RP: Reddish Purple, PG: Purple Gray.

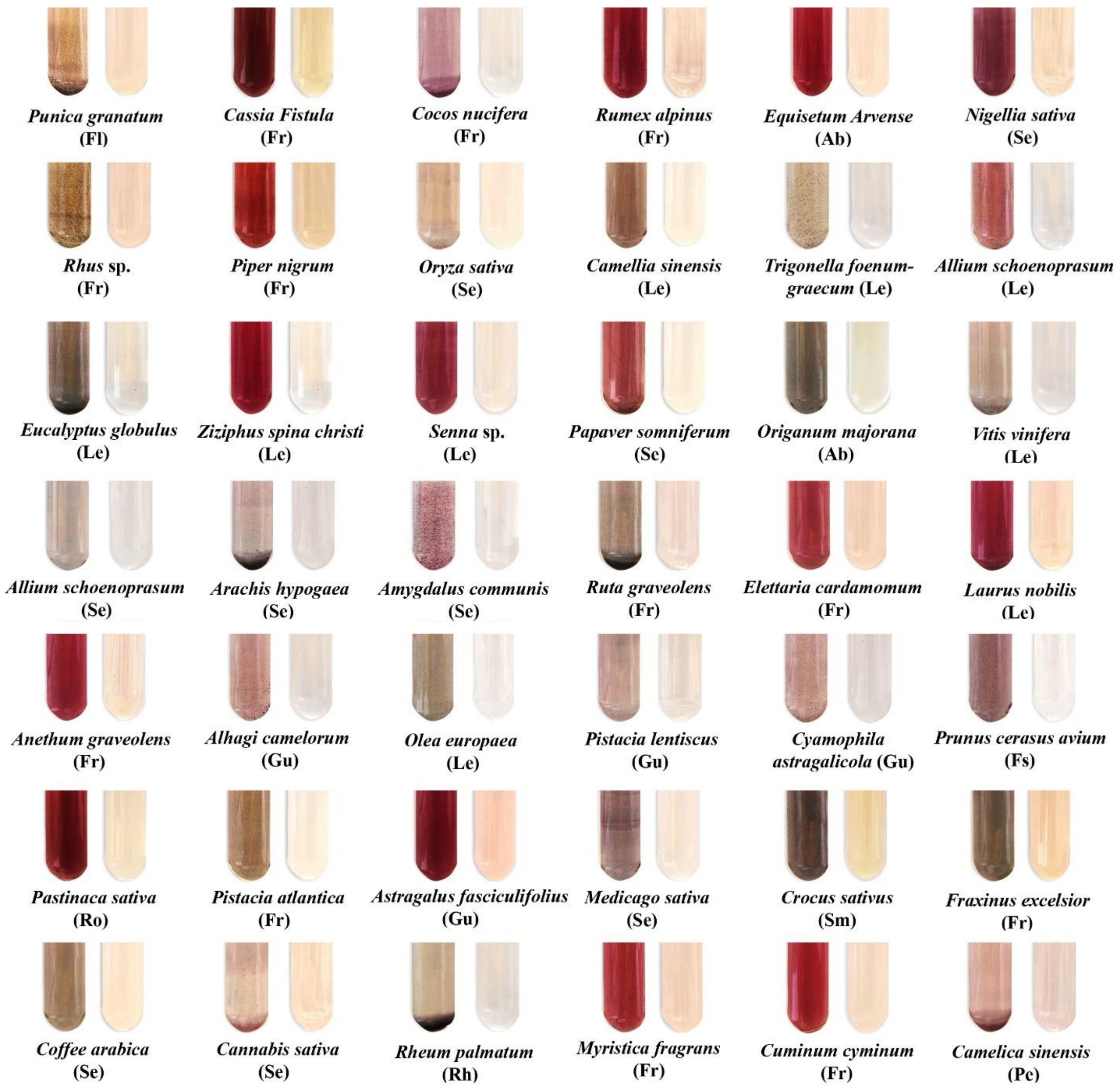
Punica granatum (Fl)
Cassia Fistula (Fr)
Cocos nucifera (Fr)
Rumex alpinus (Fr)
Equisetum Arvense (Ab)
Nigellia sativa (Se)
Rhus sp. (Fr)
Piper nigrum (Fr)
Oryza sativa (Se)
Camellia sinensis (Le)
Trigonella foenum-graecum (Le)
Allium schoenoprasum (Le)
Eucalyptus globulus (Le)
Ziziphus spina christi (Le)
Senna sp. (Le)
Papaver somniferum (Se)
Origanum majorana (Ab)
Vitis vinifera (Le)
Allium schoenoprasum (Se)
Arachis hypogaea (Se)
Amygdalus communis (Se)
Ruta graveolens (Fr)
Elettaria cardamomum (Fr)
Laurus nobilis (Le)
Anethum graveolens (Fr)
Alhagi camelorum (Gu)
Olea europaea (Le)
Pistacia lentiscus (Gu)
Cyamophila astragalicola (Gu)
Prunus cerasus avium (Fs)
Pastinaca sativa (Ro)
Pistacia atlantica (Fr)
Astragalus fasciculifolius (Gu)
Medicago sativa (Se)
Crocus sativus (Sm)
Fraxinus excelsior (Fr)
Coffee arabica (Se)
Cannabis sativa (Se)
Rheum palmatum (Rh)
Myristica fragrans (Fr)
Cuminum cyminum (Fr)
Camelica sinensis (Pc)

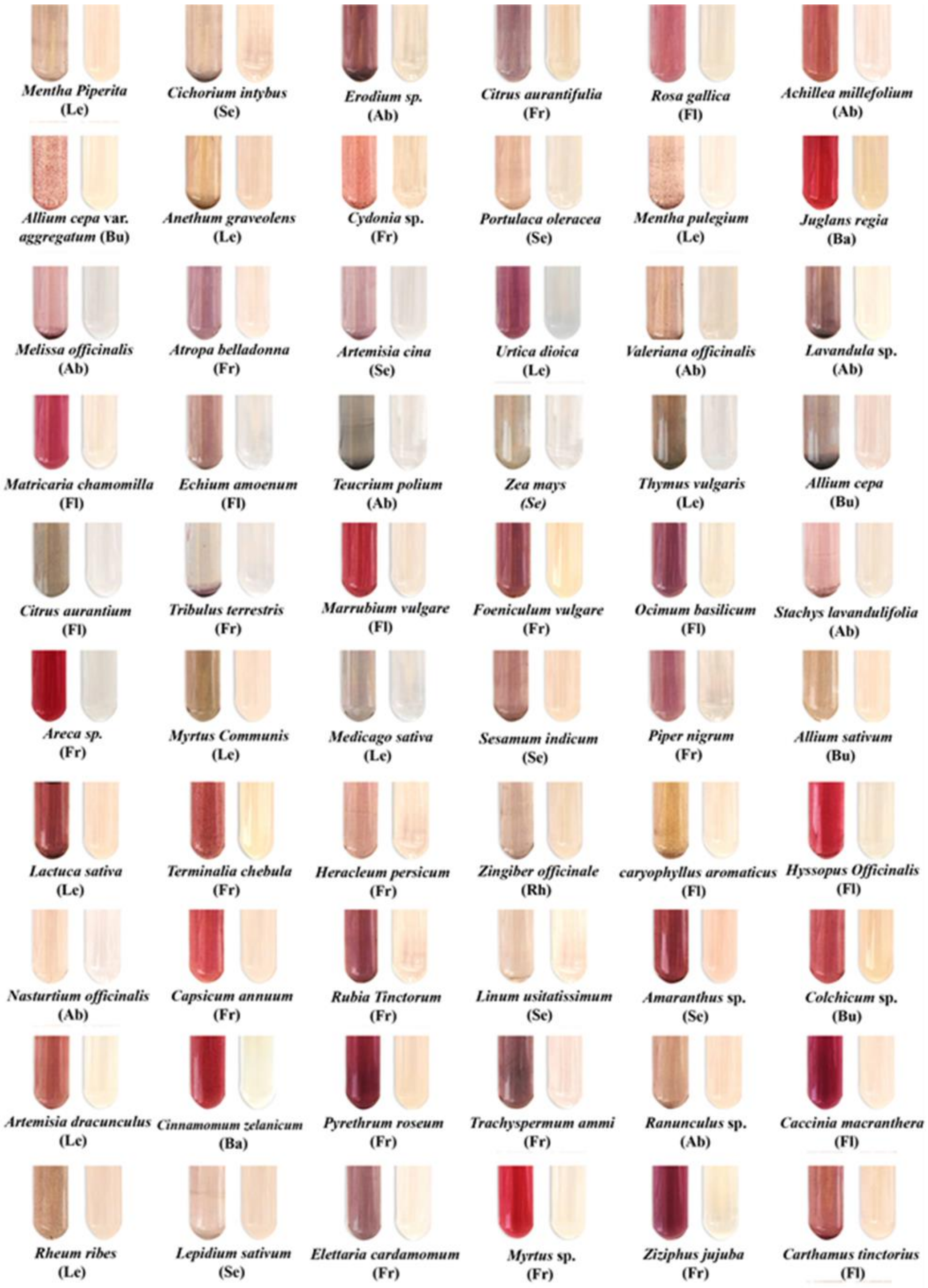

Figure S1. Pictorial results of biosynthesis of colloidal AuNPs by water extracts of 102 medicinal plant parts used in folkloric medicine of Middle East countries. Each couple of tubes is representative of its related plant sample. In all samples, the left tubes contain regenerated Au^{3+} ions in the color spectrum of pale-yellow, gray, violet, and red; while, the right tubes contain control blanks (received deionized water). Scientific names and abbreviations of used plant parts are implanted below the images as Ab: above-ground parts, Ba: bark, Bu: bud, Fl: flower, Fr: fruit, Fs: fruit stalk, Gl: glumes, Le: leaf, Pe: petioles, Rh: rhizome, Ro: root, Se: seeds, Sm: flower stamen and Wh: whole plant.
